# A case–control study of visual, auditory and audio–visual sensory interactions in children with autism spectrum disorder

**DOI:** 10.1167/jov.21.4.5

**Published:** 2021-04-08

**Authors:** Anthony M. Norcia, Azalea Lee, Wesley J. Meredith, Peter J. Kohler, Francesca Pei, Stephanie A. Ghassan, Robin A. Libove, Jennifer M. Phillips, Antonio Y. Hardan

**Affiliations:** 1 Department of Psychology, Stanford University, Stanford, CA, USA; 2 Department of Psychiatry and Behavioral Sciences, Stanford University, Stanford, CA, USA; 3 Department of Psychology, York University, Toronto, ON, Canada; 4 Centre for Vision Research, York University, Toronto, ON, Canada; 5 Department of Psychiatry and Behavioral Sciences, Stanford University, Stanford, CA, USA

**Keywords:** autism spectrum disorder, attention deficit hyperactivity disorder, sensory processing, visual evoked response, auditory evoked response, audio–visual interaction

## Abstract

To assess the relative integrity of early visual and auditory processes in autism spectrum disorder (ASD), we used frequency-tagged visual and auditory stimulation and high-density electroencephalogram recordings of unimodal and dual-modality responses in a case–control design. To test for the specificity of effects on ASD, we recorded from a smaller group of children with attention-deficit hyperactivity disorder (ADHD). Horizontal 3 cycle per degree (cpd) gratings were presented at 5 Hz, and a random stream of /ba/, /da/, /ga/ syllables was presented at 6 Hz. Grating contrast response functions were measured unimodally and in the presence of a 64-dB auditory input. Auditory response functions were measured unimodally and in the presence of a 40% contrast grating. Children with ASD (*n* = 34) and ADHD (*n* = 13) showed a common lack of audio–visual interaction compared to typically developing children (*n* = 40) when measured at the first harmonic of the visual stimulus frequency. Both patient groups also showed depressed first harmonic responses at low contrast, but the ADHD group had consistently higher first-harmonic responses at high contrast. Children with ASD had a preferential loss of second-harmonic (transient) responses. The alteredtransient responses in ASD are likely to arise very early in the visual pathway and could thus have downstream consequences for many other visual mechanisms and processes. The alteration in audio–visual interaction could be a signature of a comorbid phenotype shared by ASD and ADHD, possibly due to alterations in attentional selection systems.

## Introduction

Alterations in sensory processing have been reported in a range of developmental disorders, including autism spectrum disorder (ASD) (Beker, Foxe, & Molholm, 2018; Dellapiazza, Vernhet, Blanc, Miot, Schmidt, & Baghdadli, 2018; Kanner, 1943; Marco, Hinkley, Hill, & Nagarajan, 2011; Ocak, Eshraghi, Danesh, Mittal, & Eshraghi, 2018; Robertson & Baron-Cohen, 2017) and attention deficit hyperactivity disorder (ADHD) (Bijlenga, Tjon-Ka-Jie, Schuijers, & Kooij, 2017; Fuermaier et al., 2018; Ghanizadeh, 2011; Kim, Banaschewski, & Tannock, 2015; Lau-Zhu, Fritz, & McLoughlin, 2019). These alterations include both hyper- and hyposensitivity to simple sensory inputs devoid of social, emotional, or linguistic/cognitive content and suggest that alterations may be present in the primary sensory areas or their thalamic inputs. Addressing the anatomical locus of sensory processing alterations is best accomplished through neural measurements. Consistent with this observation, the neural bases of sensory processing alterations in ASD and ADHD have begun to be understood, primarily through the use of sensory evoked potentials/magnetic fields generated in response to simple stimuli such as gratings and tones.

In ASD, visual evoked potentials (VEPs) to simple visual patterns, such as gratings or checkerboards, differ between persons with ASD and typically developing controls in a number of studies. In the majority of these studies, evoked response amplitude is reduced (Boeschoten, Kenemans, van Engeland, & Kemner, 2007; Jemel, Mimeault, Saint-Amour, Hosein, & Mottron, 2010; Kornmeier, Worner, Riedel, Bach, & Tebartz van Elst, 2014; Kovarski, Thillay, Houy-Durand, Roux, Bidet-Caulet, Bonnet-Brilhault, Batty, 2016; Milne, Scope, Pascalis, Buckley, & Makeig, 2009; Pei, Baldassi, & Norcia, 2014; Siper et al., 2016; Vilidaite et al., 2018; Weinger, Zemon, Soorya, & Gordon, 2014). In other studies, evoked response amplitude is larger for persons with ASD (Frey, Molholm, Lalor, Russo, & Foxe, 2013; Takarae, Sablich, White, & Sweeney, 2016; Vilidaite et al., 2018; Vlamings, Jonkman, van Daalen, van der Gaag, & Kemner, 2010) at least for some stimulus conditions or participant groups. Finally, several studies have reported response components that are not altered (Constable, Gaigg, Bowler, & Thompson, 2012; Kovarski et al., 2016). The results of these studies are summarized in Table 1.

**Table 1.** Summary of visual and auditory evoked response studies. *Notes:* ASD = autism spectrum disorder; cpd = cycles per degree; ERP = evoked response potential; M100 = 100-ms auditory response; M200 = 200-ms auditory response; MEG = magnetoencephalography; PDD = pervasive developmental disorder; SPL = sound pressure level; STG = superior temporal gyrus; TD = typically developing; VESPA = visual evoked spread spectrum analysis.

Visual							
Study	Year	Age (years)	Diagnosis	Spatial	Contrast	Temporal	Results
Boeschoten et al.	2007	10	PDD high functioning	0.75, 1.5, and 6 cpd square wave	100%	Onset	Reduced amplitude ERP for 6 cpd
Milne et al.	2009	13	ASD	0.5, 1, 4, and 8 cpd sine wave	High	Onset	Reduced amplitude for 4 and 8 cpd
Jemel et al.	2009	26	ASD and Aspberger	0.8 and 8 cpd sine wave	4%, 8%, 32%, 90%	2-Hz reversal	Reduced gain for N80 at 8 cpd, lower amplitude at high-contrast 0.8 cpd at P100
Vlamings et al.	2010	4	ASD	0.75 and 6 cpd square wave	100%	Onset	Larger 6-cpd grating response in ASD
Constable et al.	2012	36–49	ASD	0.83° checks	10%, 90%	3-Hz reversal	No effect
Frey et al.	2013	11	ASD	0.8° checks	—	1-Hz reversal, VESPA	Larger amplitude for peripheral but not central fixation in ASD
Kornmeier et al.	2014	40	Aspberger	0.6° and 1.2° checks	High	Sudden-onset oddball	Reduced amplitude standards and oddballs for both check sizes
Pei et al.	2014	9	ASD	2- to 30-cpd sine wave	80%	7.5-Hz reversal	Reduced amplitude 2F for 5–17 cpd
Weinger et al.	2014	8	ASD	Isolated checks	1%–32%	12.5-Hz onset	Reduced amplitude trend at higher contrasts
Takarae et al.	2016	20	ASD high functioning	2-cpd sine wave	5%–90%	3.76-Hz on/off	Larger power in ASD than TD
Kovarski et al.	2016	22 and 15	ASD	1.9° × 1.5° checks	High (?)	1-Hz reversal	No effect N75, reduced amplitude P100
Siper et al.	2016	6	ASD	0.32° checks	100%	1-Hz reversal	Reduced amplitude early components
Vilidaite et al.	2018	23 and 9	ASD	0.5-cpd sine wave, white noise	2%–64%, 0%–50%	7-Hz on/off; 5-Hz on/off	Mixed: larger/reduced amplitudes

**Table 1.** Continued.

Auditory							
Study	Year	Age (years)	Diagnosis	Waveform (Tones, Hz)	Intensity	Temporal	Results
Roberts et al.	2010	10	ASD	200, 300, 500, and 1000	45-dB sensation level	300-ms burst	Delayed M100 in ASD
Gage et al.	2003	11–14	ASD	250 and 1000	40-dB sensation level	400-ms burst	Elongation of M100 in ASD
Edgar et al.	2014	9 and 13	ASD	1000 and 2000	45-dB sensation level	50-ms pulse pairs	Delayed M50, more frequently missing M100 in ASD
Brandwein et al.	2015	11	ASD	1000	75-dB SPL	60-ms burst	Reduced amplitude N1b
Edgar et al.	2015	10	ASD	500 and 1000	45-dB sensation level	300-ms burst	Reduced amplitude M200 in right STG
Port et al.	2016	8	ASD	200, 300, 500, and 1000	45-dB sensation level	300-ms burst	Delayed M100 in ASD
Stephen et al.	2017	3.5	ASD	800	60-dB SPL	100-ms burst	Delayed MEG components in ASD

Similarly, auditory evoked fields and potentials to simple stimuli such as tones have also been found to be altered in ASD, with delays of components being a common observation (Bomba & Pang, 2004; Brandwein, Foxe, Butler, Frey, Bates, Shulman, & Molhome, 2015; Edgar et al., 2014; Edgar et al., 2015; Gage, Siegel, & Roberts, 2003; Port et al., 2016; Roberts et al., 2010; Stephen, Hill, Peters, Flynn, Zhang, & Okada, 2017). The results of these studies are summarized in Table 1.

Beyond unimodal sensory alterations in ASD, there are many reports of altered audio–visual (A/V) interactions, particularly for language-related stimuli, but also between low-level auditory and visual sensory stimuli measured behaviorally (Baum, Stevenson, & Wallace, 2015a; Baum, Stevenson, & Wallace, 2015b). Facilitative A/V neural interactions between an auditory tone and visual colored-disk stimulus measured both behaviorally and with event-related potentials are reduced in ASD participants, as is behavioral facilitation in the form of decreased simple reaction times (Brandwein, Foxe, Butler, Russo, Altschuler, Gomes, & Molhome, 2013; Brandwein et al., 2015). A recent functional magnetic resonance imaging (fMRI) study found that auditory stimuli fail to downregulate activation in the early visual cortex of children with ASD relative to typically developing controls (Keehn, Sanchez, Stewart, Zhao, Grenesko-Stevens, Keehn, & Müller, 2017).

Here, we sought evidence for altered unimodal sensory responses and altered A/V interactions through the use of frequency-tagged steady-state visual evoked potentials (SSVEPs) and steady-state auditory evoked potential (SSAEPs). Steady-state evoked responses involve the presentation of stimulus trains in which a parameter such as contrast or loudness varies periodically. This periodic stimulus drive generates a periodic brain response at the modulation frequency and its harmonics. Because the possible response frequencies are known exactly and the response itself is strictly confined to harmonics of the stimulation frequency, it is possible to extract the driven responses from the background experimental noise through sensitive spectral analysis procedures that have high signal-to-noise ratio (SNR) (Norcia, Appelbaum, Ales, Cottereau, & Rossion, 2015). Moreover, by presenting auditory and visual stimuli at different temporal frequencies, spectral analysis can separate responses evoked by the two sensory modalities, even when the auditory and visual stimuli are presented simultaneously. By varying the intensity of one of the unimodal inputs, its effect on the response of the other modality can be measured directly. This method has the advantage that unimodal and cross-modal responses can be measured simultaneously, controlling for state and experimental noise variations that can contaminate additivity-failure indices of A/V interaction that rely on summing separate unimodal measurements to estimate cross-modal interaction.

In addition to measuring A/V interactions, we also measured the increase in sensory responses as a function of stimulation intensity and determined their intrinsic response dynamics. The form of the stimulus response function is of theoretical interest, and measurements of stimulus–response functions are more informative than single measurements at a single supra-threshold level. Specifically, ASD sensory processing differences have been interpreted to be the result of alterations in the balance of excitation and inhibition (E/I) (Rosenberg, Patterson, & Angelaki, 2015; Rubenstein & Merzenich, 2003; Yizhar et al., 2011). E/I imbalance theory suggests that the typically observed saturation visual contrast response function at high contrasts may be altered if the E/I balance is shifted toward excitation (Rosenberg et al., 2015). We test this prediction by measuring sensory response magnitude as a function of a wide range of sensory input intensities.

Using the SSVEP, we have previously found evidence of a selective loss of transient visual activity in children and adults with ASD and in a fruit-fly ASD model (Vilidaite et al., 2018). Transient versus sustained activity in the SSVEP was assessed through an analysis of different response harmonics. Based on symmetry considerations, the first harmonic was used as an assay of sustained activity and the second harmonic as an assay of transient activity (McKeefry, Russell, Murray, & Kulikowski, 1996). As our previous results suggest that a fundamental alteration in the temporal dynamics of the visual response is present in ASD, another goal of the present study was to replicate this finding in a larger sample of children. Finally, because sensory processing alterations are present in many developmental disorders, we compared sensory evoked responses in children with ASD to a control sample of children with ADHD. ADHD is a particularly salient developmental disorder for comparison, given that there is considerable comorbidity between ASD and ADHD (Antshel & Russo, 2019; van der Meer, Oerlemans, van Steijn, Lappenschaar, de Sonneville, Buitelaar, Rommelse, 2012). By comparing the evoked responses in the two disorders, we sought to determine whether any alterations observed were specific to the child's clinical diagnosis. We replicated the result of a relative loss of transient visual activity in children with ASD that we reported previously and found that it is specific to ASD. We also found that A/V interactions are weakened in both ASD and ADHD.

## Methods

We report here the results of a case–control study of sensory processing in children with ASD and typically developing (TD) controls. Additionally, we provide data from a comparison group of children with ADHD to assess the specificity of effects to ASD.

### Participants

Fifty-six children between the ages of 4 and 9 years with ASD (44 male; mean age, 6.30 years; *SD* = 1.69), 23 children with ADHD (21 male; mean age, 7.39 years; *SD* = 1.59), and 51 TD children (41 male, mean age, 6.06 years; *SD* = 1.50) were initially enrolled. All participants who completed the study had normal or corrected vision, normal hearing, and no history of severe neurological problems based on a review of their medical history. Three children in the ASD group and four children in the ADHD group on medications were determined to be on a stable dosage for at least 1 week prior to the electroencephalogram (EEG) recordings. Children whose parents expressed concern about visual ability within the TD group were tested using a 20-foot optotype visual acuity chart. Seventeen children with ASD did not meet inclusion or exclusion criteria. Of these, 11 were excluded because we were unable to successfully record EEGs, and five were withdrawn from the study by the parent/guardian, resulting in 34 ASD children being included in the analysis (28 male; mean age, 7.0; *SD* = 1.82). Approaching children with autism is challenging in general and especially challenging when a stranger wants to put an unfamiliar appliance on the child's head. Our anecdotal sense is that failures were more often due to not actually placing the net or the child not wanting to proceed with testing, rather than poor data quality after the net was placed. Nine children with ADHD did not meet inclusion or exclusion criteria, one of whom was excluded because of an inability to record EEGs successfully. One additional participant was withdrawn by the parent/guardian, resulting in 13 children whose data were analyzed (11 male; mean age = 8.2; *SD* = 1.3). Four children from the TD group did not meet inclusion or exclusion criteria, and seven children were withdrawn from the study, resulting in 40 participants included in the analyses versus those submitted for analysis (32 male; mean age, 6.3; *SD* = 1.4). The research conformed to the tenets of the Declaration of Helsinki and was approved by the Stanford University institutional review board. The parent or legal guardian of each participant provided informed consent. When developmentally appropriate, participants at least 7 years old or older additionally provided their assent.

### Participant assessment

All but one of the participants completed the Stanford Binet Intelligence Scales, Fifth Edition (SB-5) assessment of cognitive functioning (Roid, 2003). ASD and ADHD diagnoses by a clinician were required before enrollment and then confirmed by assessors who administered the Autism Diagnostic Observation Schedule-2 (ADOS-2) (Lord, Rutter, DiLavore, Risi, Gotham, & Bishop, 2012) and Autism Diagnostic Interview- Revised (ADI-R) (Le Couteur, Lord, & Rutter, 2003) for children in the ASD group (30/34 participants), or the Kiddie Schedule for Affective Disorders and Schizophrenia for School-Aged Children, Present and Lifetime Version (K-SADS-PL) (Kaufman et al., 1997) for children in the ADHD group (all participants). The ADOS-2 is a standardized observational child assessment used to diagnose autism spectrum disorders, whereas the ADI-R is a comprehensive parent/caregiver interview that assesses a child's language, social interactions, and restrictive, repetitive behaviors in the past and present. The K-SADS-PL is a semi-structured diagnostic interview assessing the presence of psychopathology in past and present for children and adolescents. TD children were screened for any psychopathology using the Child Behavior Checklist (Aschenbach & Ruffle, 2000). Participants scoring high on any of the symptom domains, such as anxiety or mood, were excluded from the study after an additional parent interview with a clinician to confirm the presence of psychiatric disorder. Additional details of the inclusion and exclusion criteria and assessments are provided in the Supplementary Materials S1. Individual participant demographics, intelligence quotient (IQ), and ADOS scores are provided in Supplementary Materials S2.

### Audio–visual stimulation

Visual and auditory displays were generated using in-house software written in Objective C running on an iMac computer (Apple Inc., Cupertino, CA). Visual and auditory stimuli were presented simultaneously and separately at unique temporal frequencies to elicit distinct steady-state visual and auditory potentials (SSVEPs and SSAEPs). The temporal frequencies were chosen so that the frequency-tagged evoked responses would be measured against a similar level of background EEG noise while still evoking reliable steady-state responses (Alaerts, Luts, Hofmann, & Wouters, 2009; Skoczenski & Norcia, 2002). Children viewed horizontal 3.0-cpd sine-wave gratings that were sine-wave contrast modulated at 5 Hz in onset/offset mode, and they listened to repetitions of “/ba/, /da/, /ga/” syllables produced by an artificially synthesized human voice at 6 Hz. The sound files were converted to voltages to drive the speaker via a 16-bit digital-to-analog converter (National Instruments, Austin, TX). The stimulation rate was sufficiently low that speech sounds were still intelligible. The temporal order of the syllables was randomized. The visual stimuli were presented on a Sony PVM-2541 organic light-emitting diode monitor (1920 × 1800-pixel resolution, 60-Hz vertical refresh rate, 50-cd/m^2^ mean luminance; Sony Corporation, Tokyo, Japan). The viewing/listening distance was 100 cm, resulting in a 17 × 17-degree subtended visual angle. Auditory stimuli played from a Yamaha MSP5 Studio speaker (Yamaha Corporation, Hamamatsu, Japan) located directly above and centered on the display monitor.

**Figure 1.**
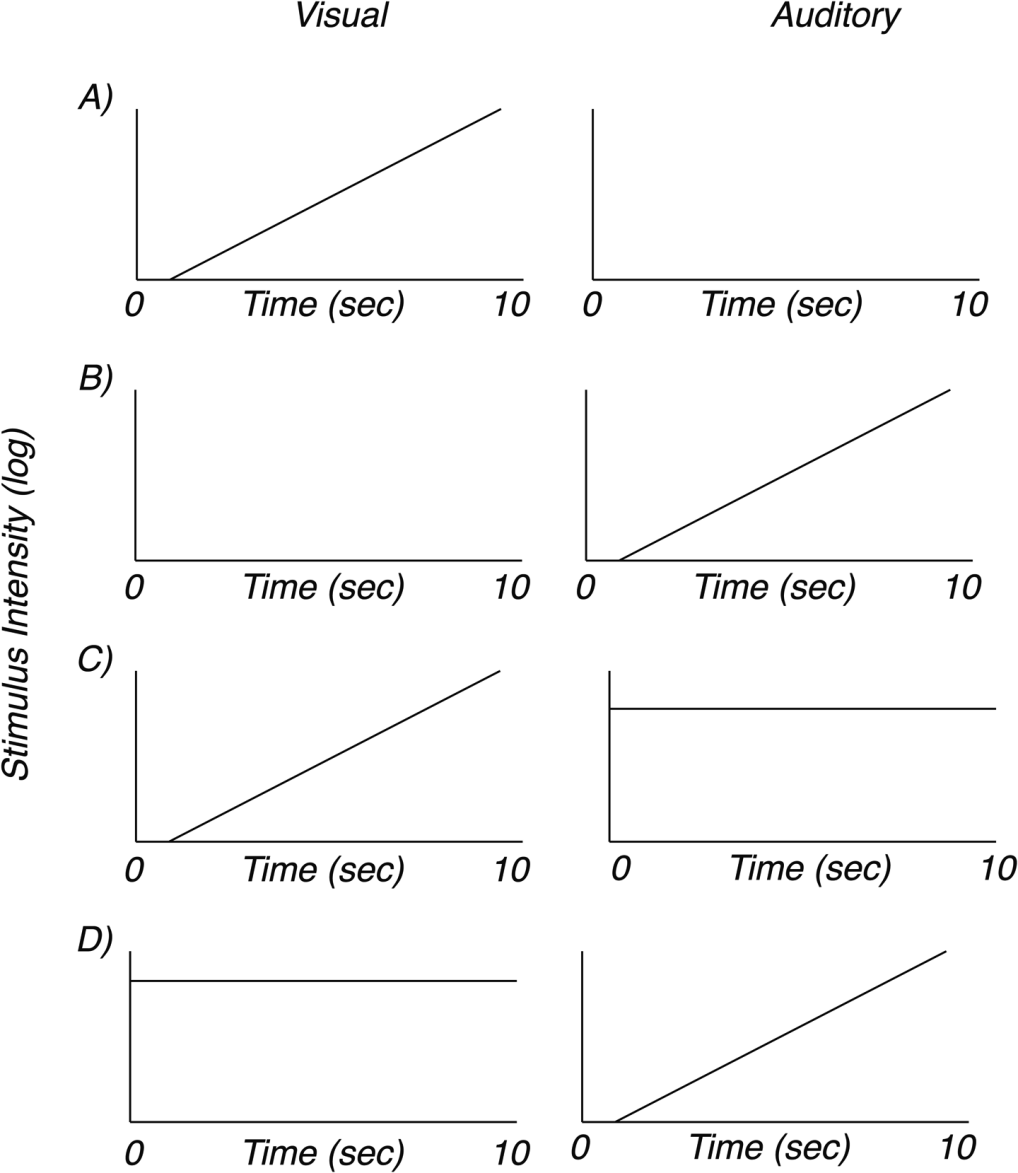
Stimulus schematic. (A) The visual-only condition was comprised of a visual contrast sweep with no auditory input. (B) The auditory-only condition was comprised of a loudness sweep with no visual input. (C) Swept visual input with constant high-level auditory input. (D) Swept auditory input with constant high-level visual input. Visual stimuli were presented at 5 Hz, auditory stimuli were presented at 6 Hz. Generic log stimulus intensity values are used to label the *y*-axis; see Methods for details.

The stimulus conditions are illustrated schematically in Figure 1. Participants viewed four conditions: *visual sweep alone*, *auditory sweep alone*, *visual sweep with auditory stimulation*, and *auditory sweep with visual stimulation*. For the *visual sweep alone* stimulus (Figure 1A), the contrast of the grating was swept from 0.8% to 80% contrast in 10 equally spaced logarithmic steps, each lasting 1 second. In the *auditory sweep alone* condition (Figure 1B), sound intensity was similarly swept in 10 1-second logarithmic steps from 0.002 to 0.2 (40 dB) in voltage units within our in-house presentation software, resulting in sound pressure levels of ∼30 to 70 dB at the listener's position. For the *visual sweep with auditory stimulation* condition (Figure 1C), participants viewed the same contrast sweep described for the *visual alone* condition while simultaneously receiving a fixed auditory stimulus at an intensity of 0.1 native units (∼64 dB). The *auditory sweep with visual stimulation* condition (Figure 1D) presented the same auditory sweep described for the *auditory sweep alone* condition while participants also viewed a fixed visual stimulus at 40% contrast. Each trial began with a 1-second “prelude” stimulus that was identical to the stimulation presented during the first second of the actual trial. The prelude was designed to allow the brain response to achieve steady-state before the actual data collection began.

### Experimental procedure

During the recording, participants were instructed to fixate on a white cross centered on the screen, regardless of presented condition. A research assistant sat beside the participant, monitoring their fixation and providing verbal encouragement to the child in between trials, as well as feedback to another researcher monitoring the EEG recording from a partitioned computer setup running the data acquisition software. One trial from each of four conditions was presented during a single randomized block of stimuli. Breaks were given as needed between blocks. Most participants completed ten blocks, resulting in 10 trials per condition (40 10-second trials total).

### EEG data acquisition and signal processing

Data were collected using 128-channel HydroCel Geodesic Sensor Nets and a Net Amps 400 system (Electrical Geodesics, Inc., Eugene, OR). Display software “tagged” conditions and trials with serial digital triggers with millisecond accuracy. Data were then filtered using a 0.3- to 50-Hz passband during export. In-house signal processing software located sample-by-sample instances where channels exceeded a threshold ranging from 30 to 260 μV on more than 15% of samples. Thresholds were set on a case-by-case basis, and the median threshold was 50 μV. These noisy channels were then replaced by an interpolated average of their six closest neighbors. After channel replacement, all EEG channels were re-referenced from Cz to the common average of all channels. Epoch-by-epoch analysis at the scale of 1 second then removed samples exceeding thresholds ranging between 60 and 520 μV, depending largely on participant group and movement artifacts. The rates of artifact rejection were similar across the three participant groups. The median fractions of usable 1-second data bins over all four stimulus conditions were 0.85 for the TD group, 0.83 for the ASD group, and 0.83 for the ADHD group.

### Analysis procedures

#### Spectral analysis

Because visual and auditory stimuli were tagged at 5 Hz and 6 Hz, respectively, Fourier analysis (Regan & Cartwright, 1970; Regan & Heron, 1969) was used to isolate responses to either sensory stimulus, at the harmonics of the auditory and visual stimulation frequencies. In our sweep paradigm, stimulus values were updated for every 1-second bin, so each bin in our analysis in a given trial type was tied to a distinct set of stimulus parameters. The amplitude and phase of the SSVEPs were extracted using a discrete Fourier transform calculated on non-overlapping 1-second bins during the 10-second trials. Real and imaginary components of the SSVEPs at the first four harmonics of the stimulus frequency were calculated.

### Dimensionality reduction

Because the steady-state evoked response phase is constant over repeated trials of the same stimulus, we used a spatial filtering approach (Dmochowski, Greaves, & Norcia, 2015) called reliable components analysis (RCA) to reduce the dimensionality of the 128-channel EEG to a single component that reflected the activity that was maximally reliable in terms of amplitude and phase. This optimization criterion is consistent with the assumption that the SSVEP/SSAEP and the background EEG and other experimental noise sources are additive. RCA is based on a generalized eigenvalue decomposition of the cross-trial covariance matrix. RCA is similar to principal components analysis, except that it maximizes cross-trial similarity rather than dimensions of maximal variability.

The real and imaginary values for each 1-second analysis bin across the 128 sensors and across trials and participants served as the input data for the RCA. Reliable components were derived separately to extract visual versus auditory response components that were expected to have different underlying generators and thus different scalp topographies. We performed two RCAs, each based on different subsets of the data. The visual RCA was based on the first four harmonics of the visual stimulus frequency (5 Hz) in the *visual sweep alone* and *visual sweep with auditory stimulation* conditions. The auditory RCA was based on the first four harmonics of the auditory stimulus frequency (6 Hz) in the *auditory sweep alone* and *auditory sweep with visual stimulation* conditions. We describe the individual harmonic components using the following notational convention: The visual stimulus is labeled as the first frequency (F1) and the auditory stimulus as the second frequency (F2). The harmonics of the visual response are denoted as 1F1, 2F1, 3F1, and 4F1, and the harmonics of the auditory response are denoted as 1F2, 2F2, 3F2, and 4F2. The spatial weights of the first RCA component for each stimulus modality reflect the scalp topography of the corresponding evoked response, along with spectral amplitudes at each harmonic for each 1-second bin of the stimulation trials. Our analyses focused on 1F1 and 2F1 data from the first, most reliable component generated by the visual RCA and 1F2 and 2F2 data from the first, most reliable component generated by the auditory RCA.

### Group-average response functions

After projecting the bin-level data through the RCA component weights, group-level averages across sweep trials for each condition and across participants were generated using phase-sensitive averaging. First, the real and imaginary coefficients for a given harmonic were averaged across participants, and the amplitude and phase were computed from the result. The vector averages were computed separately for each of the 10 bins spanning the 10-second trials.

Prior to the group-level statistical analysis, we converted the vector-valued bin data (amplitude and phase) to scalar values (amplitude only) so that we could use conventional mixed-effects modeling to assess group-level and bin-level effects. Preliminary analyses indicated that there were no clear effects of group membership on response phase. Scalar amplitude values were computed as the magnitude of the projection of each participant's response vector onto the group vector average (Hou, Gilmore, Pettet, & Norcia, 2009). The magnitudes of these projections were then used to compute the mean amplitude and standard error for each condition and to conduct the linear mixed-effects analysis described below. Note that the mean of these projected amplitudes is the approximately same as the amplitude of the vector average. The projection procedure is useful because it preserves the observed robust phase consistency across participants with associated SNR improvements that would not occur if amplitude means and errors and corresponding tests were simply computed from individual participant amplitudes.

### Statistical analysis

To determine whether measurable evoked responses were present at the group level, we computed one-sided *t*-tests that tested whether the distribution of amplitudes was different from zero for each response bin. To determine the effects of GROUP (ASD, ADHD, TD) and stimulus value within each of the 10 bins associated with different swept stimulus values (BIN) and their possible interaction, we performed linear mixed-effects analysis (LMEA) using the lme4 package (Baayen, Davidson, & Bates, 2008) in R (R Core Team, 2014). We used an analysis of variance to test for significance as implemented using the lmerTest package in R (Kuznetsova, Brockhoff, & Christensen, 2013). LMEA was performed separately for data from each combination of response component (visual RC1, auditory RC1), harmonic (first and second), and condition, with BIN as a within-participant factor and GROUP as a between-participant factor. The model tested for main effects of BIN and GROUP, as well as for the interaction between BIN and GROUP. To test the significance of the interaction, we also compared the fit of a simpler model that left out the interaction term, using a likelihood ratio test to compare the goodness of fit of the two models. The likelihood ratio test follows a chi-square distribution, with degrees of freedom being equal to the number of additional parameters in the more complex model. If *p* < α, the more complex model significantly improves the fit of the model to the data. For all of these analyses, *p* values and denominator degrees of freedom were calculated using Satterthwaite's approximations (Kuznetsova, Brockhoff, & Christensen, 2017).

## Results

The frequency-tagging approach makes it possible to selectively measure activity associated with both the visual and auditory stimuli, even when they are presented simultaneously. We first describe the visual responses measured at the visual response frequencies and then the auditory responses measured at their response frequencies.

### Unimodal visual contrast response function

**Figure 2.**
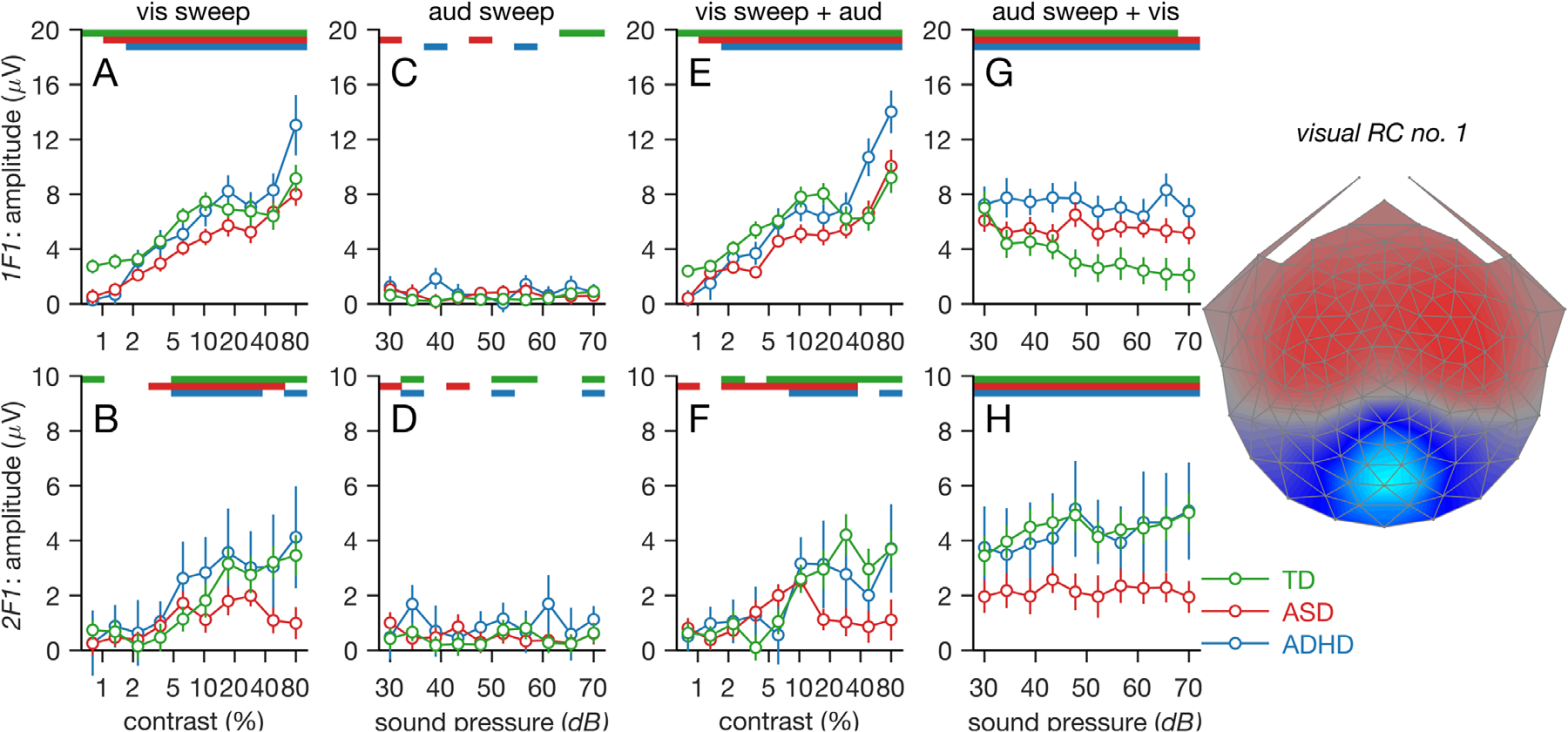
Responses at visual stimulus-related frequencies, 1F1 (top row) and 2F1 (bottom row), derived from corresponding visual RC1 (see rightmost panel for RC1 topography). Data from TD children are shown in green, ASD children in red, and ADHD children in blue. Unimodal visual responses are shown in (A) for 1F1 and in (B) for 2F1. Background EEGs at visual response frequencies during unimodal auditory stimulation are shown in (C) and (D) for 1F1 and2F1, respectively. Visual contrast response functions measured in the presence of a highly supra-threshold auditory input are shown in (E) and (F) for 1F1 and 2F1, respectively. Visual responses to a 40% contrast grating measured in the presence of a variable sound-level auditory input are shown (G) and (H) for 1F1 and2F1, respectively. Color-coded bars at the top of the response function panels indicate evoked responses significantly different than zero at the *p* < 0.05 level. See text for details.

Figures 2A and B plot SSVEP amplitude as a function of stimulus contrast at the first and second harmonics 1F1 and 2F1 generated at 5 and 10 Hz, respectively. Data are shown for RC1, the most reliable component, the topography of which is shown on the right side of Figure 2. RC1 is focally distributed at electrodes over early visual areas.

In the *visual sweep alone* condition, the contrast of the grating was swept from low to high, and the response functions for each participant group increased as a function of stimulus contrast (green, TD; red, ASD; blue ADHD). For comparison, data from the same harmonics (1F1 and 2F1) in the *auditory sweep alone* condition are shown in Figures 2C and 2D. Because no evoked response was expected at these response frequencies when no visual stimulus was presented, these data reflect the experimental noise level of the visual-alone measurement. The 1F1 response in the TD group is significantly above the noise level at the lowest contrast (see green bar at the top of Figure 2A), whereas those of the ASD and ADHD groups are not (see corresponding red and blue bars). The TD group response function saturates at mid-contrast levels, but the response functions generated by the ASD and ADHD groups do not.

The LMEA output is consistent with these observations. For 1F1 responses, the interaction between GROUP and BIN was significant, *F*(2, 780) = 10.77, *p* < 0.001, and adding interaction terms between BIN and GROUP improved the model fit, χ^2^(2) = 21.36, *p* < 0.001. There was also a main effect of GROUP, *F*(2, 84) = 3.60, *p* = 0.032, such that ASD had weaker responses than TD, *t*(84) = –2.51, *p* = 0.014, and marginally weaker responses than ADHD, *t*(84) = 1.83, *p* = 0.07. The monotonically increasing response functions in each group is captured by a main effect of BIN, *F*(1, 780) = 359.10, *p* < 0.001.

The 2F1 responses increase with stimulus contrast in the TD and ADHD groups, but not in the ASD group, which responds maximally at 20% to 40% contrast rather than at 80% contrast and generates overall lower amplitudes (see Figure 2B). Consistent with this, there was a significant interaction between GROUP and BIN, *F*(2, 780) = 6.56, *p* = 0.001, due to the lower responses in ASD. Adding interaction terms between BIN and GROUP improved model fit, χ^2^(2) = 13.07, *p* = 0.001. The main effect of GROUP did not reach significance, *F*(2, 84) = 2.22, *p* = 0.12, but there was a significant main effect of BIN, *F*(1, 780) = 58.60, *p* < 0.001.

### Visual contrast response function measured during auditory stimulation

The visual responses to the same sweep from low to high contrast used in the unimodal visual measurement, but recorded in the presence of a constant, highly supra-threshold auditory stimulus (∼64 dB), strongly resemble the unimodal measurements. This result indicates that there was little influence of the constant-loudness auditory stimulus on the 1F1 and 2F1 responses from early visual cortex.

**Figure 3.**
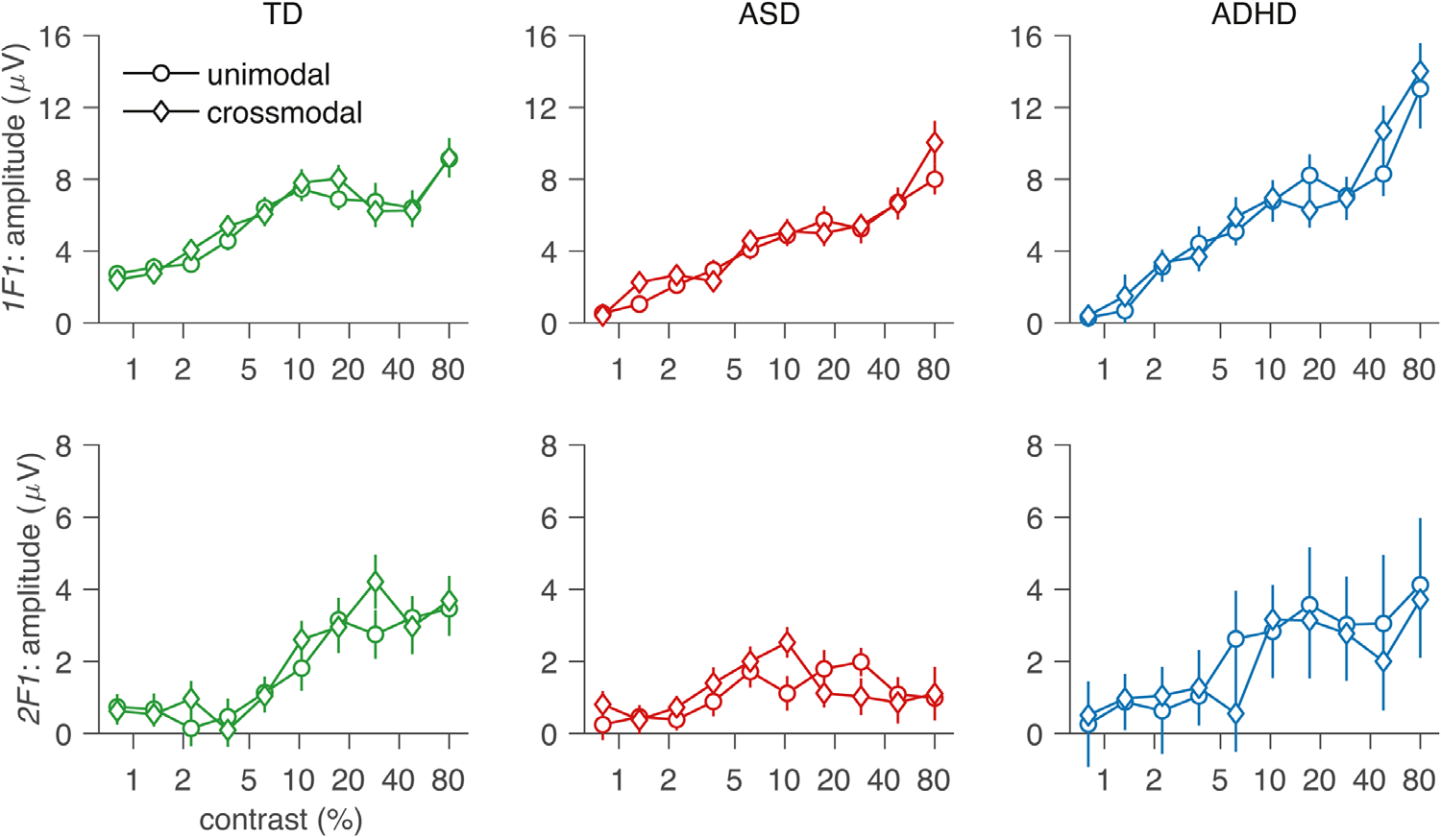
Visual contrast response function measured without (circles) and with (diamonds) a high-sound-level auditory input present. The TD group is shown in green, ASD group in red, and ADHD group in blue. Top row, 1F1; bottom row, 2F1.

That salient features of the *visual sweep alone* contrast response function were recapitulated when measured in the presence of the auditory stimulus and can be seen by comparing corresponding curves in Figures 2A and 2E for 1F1 and Figures 2B and 2F for 2F2. The similarity of responses is highlighted in Figure 3, where the data from the two conditions are plotted together for each group. The TD children had significant 1F1 responses at the lowest contrast in both unimodal and cross-modal conditions (compare Figure 2A to 2E), but this was not the case in either condition for the ASD and ADHD groups. The same panels show that the TD 1F1 response function is more saturated at high contrast than for ASD or ADHD groups and finally that the ADHD group had response amplitudes ∼40% larger than those of either TD or ASD groups at high contrast.

The LMEA of the *visual sweep with auditory stimulation* condition recapitulates the pattern of effects seen in the visual alone condition at 1F1. There was a significant interaction between GROUP and BIN at 1F1, *F*(2, 780) = 13.30, *p* < 0.001. Adding interaction terms between BIN and GROUP improved the model fit, χ^2^(2) = 26.26, *p* < 0.001. The main effect of GROUP was marginally significant, *F*(2, 84) = 2.73, *p* = 0.071, an effect that was carried by ASD having significantly weaker responses than TD, *t*(84) = –2.13, *p* = 0.04, and marginally significantly weaker responses than ADHD, *t*(84) = 1.70, *p* = 0.09. There was also a main effect of BIN, *F*(1, 780) = 374.30, *p* < 0.001.

For both the unimodal and multimodal conditions, we see a fourfold reduction of the 2F1 response at high contrast for TD relative to the other two groups (see Figures 2B, 2F, and 3). The LMEA performed on the 2F1 data from the *visual sweep with auditory stimulation* condition replicates the pattern seen in the *visual sweep alone* condition. There was a significant interaction between GROUP and BIN, *F*(2, 780) = 13.14, *p* < 0.001. Adding interaction terms between BIN and condition improved the model fit, χ^2^(2) = 25.96, *p* < 0.001. The main effect of GROUP did not reach significance, *F*(2, 84) = 1.54, *p* = 0.22, but there was a significant main effect of BIN, *F*(1, 780) = 45.25, *p* < 0.001.

### High-contrast visual response in the presence of swept auditory input

In the *auditory sweep with visual stimulation* condition, a 40% contrast grating was presented throughout the trial while an increasing-loudness auditory sweep was present starting at near threshold levels. For all three groups, the 1F1 response is at its highest amplitude when the auditory stimulus is at its lowest intensity (see leftmost data points in Figure 2G). As the intensity of the auditory input increases, 1F1 amplitude for the TD group decreases monotonically by a factor of ∼4 (Figure 2G, green curve). The decrease in 1F1 amplitude is not present for either the ASD (red curve) or ADHD (blue curve) groups. The pattern of decreasing response in the TD group and its absence manifest as a significant interaction between GROUP and BIN, *F*(2, 780) = 14.80, *p* < 0.001, and adding interaction terms between BIN and GROUP improved the model fit, χ^2^(2) = 29.17, *p* < 0.001. There was a main effect of group, *F*(2, 84) = 3.22, *p* = 0.04, carried by TD having significantly weaker responses than ADHD, *t*(84) = 2.36, *p* = 0.02, and marginally significantly weaker responses than ASD, *t*(84) = 1.68, *p* = 0.10. There was also a main effect of BIN, *F*(1, 780) = 18.49, *p* < 0.001.

Across all 10 measurements, the peak response amplitude at 1F1 is larger for the ADHD group than for either the TD or ASD group. The same pattern of elevated response amplitude at high stimulus contrast in the ADHD group can also be seen in the highest contrast bins in Figures 2A and 2E. The stimulus conditions are matched between the last two bins of the visual sweep conditions, where contrast is high, and all bins of the auditory sweep condition.

The visual responses generated at 2F1 in the *auditory sweep with visual stimulation* condition recapitulate the pattern of reduced second-harmonic activity in the ASD group when measured relative to the TD and ADHD groups. Visual responses to the constant-contrast visual stimulus are of high and constant amplitude in both TD and ADHD groups but reduced by a factor of ∼2 for all 10 measurements in the ASD group.

The overall reduction of ASD amplitude at 2F1 manifests as a significant main effect of GROUP, *F*(2, 84) = 3.64, *p* = 0.03, such that ASD had significantly weaker responses than TD, *t*(84) = –2.57, *p* = 0.01, and marginally significantly weaker responses than ADHD, *t*(84) = 1.75, *p* = 0.08. There was also a main effect of BIN, *F*(1, 780) = 9.09, *p* = 0.003, but adding interaction terms did not significantly improve model fit, χ^2^(2) = 4.55, *p* = 0.10.

The functional behavior of the 2F1 component thus differed from that of the 1F1 component. The 2F1 response was independent of loudness in the TD group, whereas the 1F1 component did depend on loudness. The two visual response components thus reflect two subsystems: one reflected in activity at 1F1, which has an A/V interaction in TD children, and another reflected in activity at 2F1, which has no such interaction.

### Unimodal auditory response function

**Figure 4.**
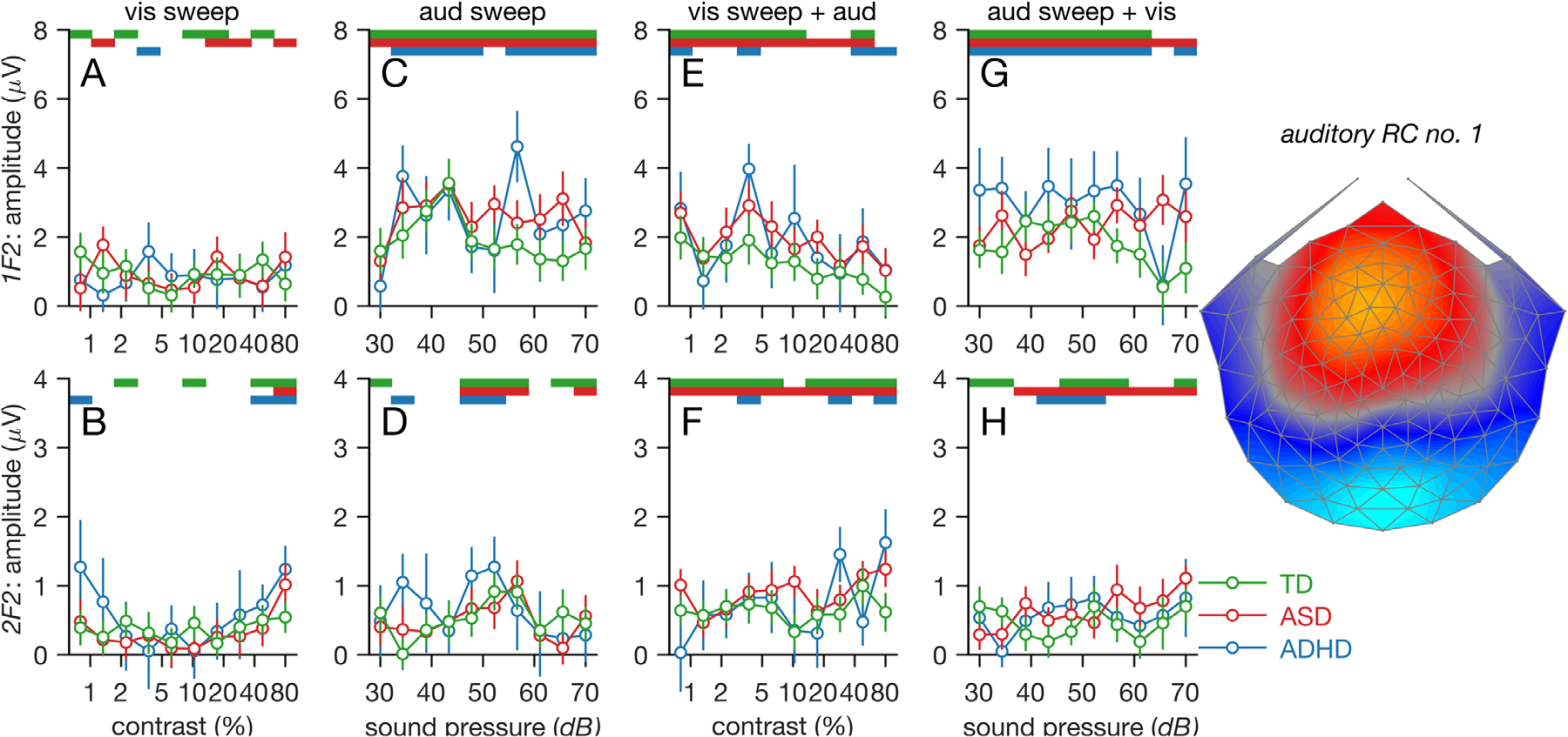
Responses at auditory stimulus-related frequencies 1F2 (top row) and 2F2 (bottom row) derived from corresponding auditory RC1 (see right panel for RC1 topography). Data from TD children are shown in green, ASD children in red, and ADHD children in blue. Background EEGs at visual response frequencies during unimodal visual stimulation are shown in panels (A) and (B) for 1F2 and 2F2, respectively. Unimodal auditory responses are shown in (C) and (D). Auditory responses to a high-sound-level input measured in the presence of a variable contrast visual input are shown in (E) and (F). Auditory loudness response functions measured in the presence of a highly supra-threshold visual input are shown in (G) and (H). Color-coded bars at the top of the response function panels plot evoked responses that are significantly different than zero at the *p* < 0.05 level. See text for details

Turning to the auditory response components measured at 6Hz (1F2) and 12 Hz (2F2), we see that the scalp topography is different from what we observed for the visual responses (Figure 4, right). Auditory responses are also closer to the experimental noise level than the visual responses. This can be seen in the background EEG levels recorded during the *visual sweep alone* condition for both 1F2 (see Figure 4A) and 2F2 (see Figure 4B). The largest auditory evoked responses at 1F2 are about a factor of 4 above the average noise level (compare Figure 4C to Figure 4A) and at most a factor of 2 larger for 2F2 (compare Figure 4D to Figure 4B). Responses at 2F2 were thus substantially weaker than those at 1F2, not reliably larger than the noise level, and not reliably different from zero (compare the number of significant responses in the bars at the top of Figures 4C and 4D), and we therefore limited further analysis to the 1F2 responses. Unlike the visual responses, the auditory 1F2 response amplitude is a non-monotonic function of stimulus loudness. The LMEA indicated that there were no main effects or interactions for the auditory-alone sweep at 1F2 (smallest *p* = 0.29).

### Auditory sound-level response function measured during high-contrast visual stimulation

**Figure 5.**
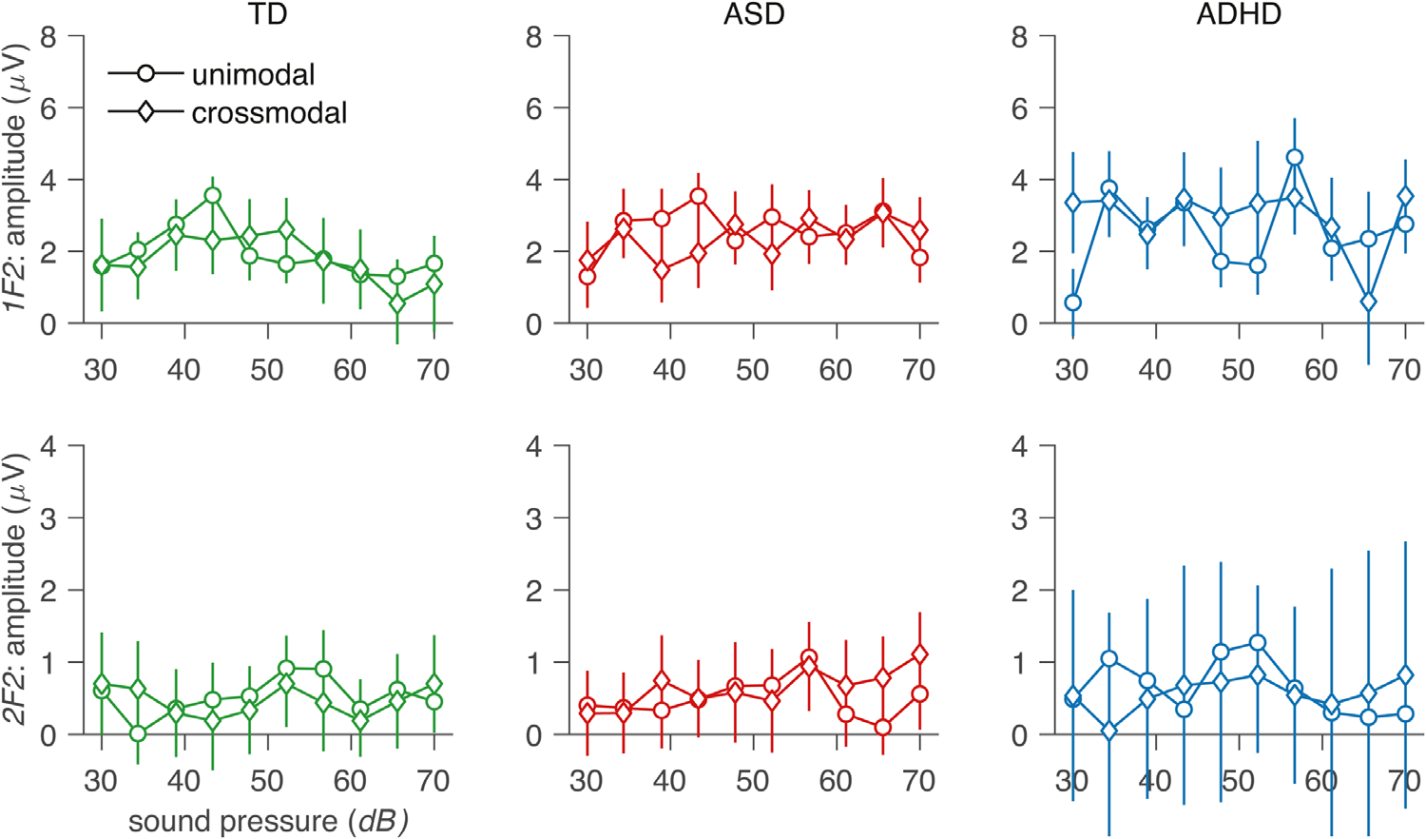
Auditory-loudness response function measured without (circles) and with (diamonds) a high-contrast visual input present. The TD group is shown in green, ASD group in red, and ADHD group in blue. Top row, 1F2; bottom row, 2F2.

In the *auditory sweep with visual stimulation* condition, the auditory sweep function was re-measured in the presence of a 40% contrast visual stimulus, and the data for 1F2 and 2F2 are shown in Figures 4G and 4H. For direct comparison, Figure 5 plots the unimodal auditory sweep function with the same function measured in the presence of a high-contrast visual stimulus separately for the three participant groups. The response functions measured with and without the visual stimulus are similar, and the LMEA for 1F2 indicates a marginally significant GROUP × BIN interaction, *F*(2, 780) = 3.12, *p* = 0.04, and adding interaction terms improved model fits, χ^2^(2) = 6.24, *p* = 0.04. There were no main effects (smallest *p* = 0.29).

### High sound-level auditory response measured in the presence of a variable-contrast visual stimulus

Figures 4E and 4F show SSAEP responses at 1F2 and 2F2, generated in response to a high-sound-level auditory stimulus (∼64 dB) during swept-contrast visual stimulation. The TD 1F2 function shows a monotonically decreasing amplitude with increasing stimulus contrast. ASD and ADHD groups both show non-monotonic functions with a peak at intermediate contrast levels, followed by a decline to a response minimum when contrast is highest, as in the TD group. There was a main effect of BIN, *F*(1, 780) = 8.78, *p* = 0.003, but no main effect of group, *F*(2, 84) = 1.76, *p* = 0.18, and adding interaction terms did not improve model fit, χ^2^(2) = 0.18, *p* = 0.92. This suggests that auditory responses were influenced by the visual stimulus in a similar way for all three groups.

## Discussion

The frequency-tagging approach allows us to measure visual and auditory responses both unimodally and under simultaneous presentation conditions where cross-modal interaction may occur. Auditory and visual evoked responses measured for ASD and ADHD children are distinct from typical responses in a number of ways, and, although some of the alterations are shared across both groups, others are specific to ASD.

### Unimodal sensory alterations

Previous VEP studies of high-contrast pattern onset or low-frequency pattern reversal responses have generally found reduced amplitudes in persons with ASD (Boeschoten et al., 2007; Kornmeier et al., 2014; Kovarski et al., 2016; Milne et al., 2009; Siper et al., 2016). By contrast, one study in young children with ASD found larger VEPs when measuring responses to the sudden onset of 6-cpd gratings (Vlamings et al., 2010).

We measured responses over a wide range of contrasts. Measurement of the contrast response function is of theoretical interest, as such measurements can shed light on the potential origins of sensory hyper- or hyposensitivity measures behaviorally. SSVEP responses rise out of the noise level close to psychophysical threshold and can thus serve as a proxy for behavioral sensitivity (Campbell & Kulikowski, 1972; Norcia, Tyler, & Hamer, 1990). Visual responsiveness at the lowest contrasts we presented was depressed in both ASD and ADHD groups relative to TD—a neural hyposensitivity rather than a hypersensitivity. This alteration is thus shared between the ASD and ADHD groups.

Neural sensory thresholds have not previously been measured and compared in these two groups, to our knowledge. A recent systematic review of 75 EEG-based studies of young adults with ASD or ADHD found no studies that have directly compared the two disorders. Clinical assessments of sensory sensitivity in ASD, either by confrontation or by questionnaires, have used supra-threshold stimulation and thus cannot speak to whether behavioral over- or under-responsivity to sensory stimulation is tied to lower or higher sensation levels, respectively (for a review of this extensive literature, see DuBois, Lymer, Gibson, Desarkar, & Nalder, 2017).

Prior psychophysical studies using static targets at higher spatial frequencies than we used have found elevated contrast sensitivity in ASD (Keita, Guy, Berthiaume, Mottron, & Bertone, 2014) or no difference (de Jonge, Kemner, de Haan, Coppens, van den Berg, & van Engeland, 2007; Koh, Milne, & Dobkins, 2010). Studies of dynamic contrast sensitivity for 6-Hz and 1-Hz flickering gratings (Bertone, Mottron, Jelenic, & Faubert, 2005) or 10-Hz flickering luminance patches (Pellicano, Gibson, Maybery, Durkin, & Badcock, 2005) found no differences between ASD and TD participants. SSVEP responses to low-contrast visual noise targets containing a wide range of spatial frequencies were equal in TD and ASD groups in our previous study (Vilidaite et al., 2018), rather than being reduced, as we found here, suggesting that the pattern of contrast sensitivity alteration in ASD/ADHD may depend on the spatial and temporal content of the stimulus.

Our measurements of the full-contrast response function also probe responsivity at high stimulation levels. Here, we found that responsivity in the children with ASD was modestly lower than in the TD children at high contrast at the first harmonic, whereas responsivity in children with ADHD was consistently higher. Elevated first-harmonic responsivity at high contrast was thus specific to the ADHD group in our measurements. Responses at the second harmonic, by contrast, were more strongly reduced in ASD but were not reduced in ADHD.

Prior studies of the VEP contrast response function have made their measurements with a range of different ages with different stimulation protocols and have yielded mixed results in terms of whether responses are larger or smaller than those of control participants. In the first study of the VEP contrast response function in ASD, event-related potentials to 2-Hz reversal in adults with ASD were found to be reduced at 8 cpd for the N80 response component and reduced at 0.8 cpd for the P100 component (Jemel et al., 2010). A subsequent study found that checkerboard 3-Hz pattern reversal responses were unaffected in adults with ASD at both 10% and 90% contrast (Constable et al., 2012). A study using isolated checks to measure SSVEP contrast response functions (Weinger et al., 2014) found no significant differences in response amplitude between children with ASD and typically developing children, although responsivity in the ASD group was consistently lower at all contrasts. One study using the SSVEP to measure responses to grating stimuli (Takarae et al., 2016) found larger responses in late adolescents with ASD compared to controls, but no change at low contrast.

Our previous study (Vilidaite et al., 2018) found a mixed pattern of larger and smaller responses, depending on age and the response harmonic measured. That study found larger first-harmonic responses at high contrast in adults with ASD and in adults with high-autistic trait profiles on the autism-spectrum quotient questionnaire (AQ) (Baron-Cohen, Wheelwright, Skinner, Martin, & Clubley, 2001), as in Takarae et al. (2016), but smaller responses at the second harmonic. The study of Takarae et al. (2016) combined first- and second-harmonic data into a composite power measure, so it is not clear whether there was a divergence of effects at the two harmonics. The only data at present that compare contrast responses to the same stimuli in both adult and immature visual systems come from our study of Nhe3 mutant flies (Vilidaite et al., 2018). The fly data suggest that both the age of the participant and the particular aspect (e.g., harmonic) of the evoked response being measured must be considered.

Responses at 1F1 in the ADHD group at high contrast levels were consistently higher than in the ASD and TD groups. This can be seen in the highest contrast bins of the response functions generated by the *visual alone sweep* and *visual sweep with auditory stimulation* conditions (Figures 2A and 2E) and in the response to a fixed, high-contrast grating in the presence of a swept auditory input (Figure 2G). Elevated responsivity at high contrast thus appears to be specific to the ADHD group in our measurements. These effects, although consistent across three independent measurements, came from a small sample of children with ADHD and thus merit a more extensive follow-up. This is especially important due to the relatively small number of reports of sensory-neural response alterations in ADHD arising from early sensory areas/mechanisms (Khaleghi, Zarafshan, & Mohammadi, 2018; Kim et al., 2015; Serrallach et al., 2016) and possible comorbid ASD effects (Lau-Zhu et al., 2019).

### Transient pathway alteration in ASD

We measured responses reflecting both linear (first harmonic) and nonlinear (second harmonic) response components and found an alteration in ASD in the latter, consistent with our prior study (Vilidaite et al., 2018). The second harmonic of the SSVEP is likely generated by transient cortical mechanisms that respond equivalently to pattern onset and offset (McKeefry et al., 1996). A previous VEP study (Frey et al., 2013) has measured linear-response components using low-contrast stimuli thought to favor responses derived from magnocellular inputs that are nominally transient. That study found no difference between ASD and neurotypical participants for centrally fixated, low-contrast patterns and concluded that there was no evidence of a magnocellular/transient-channel functional alteration. Here, we also found little effect on the linear response, but we did find a larger effect on the second harmonic, nonlinear response. We previously reported second-harmonic reductions in children with ASD over a range of spatial frequencies between 5 and 17 cpd (Pei et al., 2014). Alterations in nonlinear response components have also been reported in adults who scored high on an index of autistic traits (Sutherland & Crewther, 2010). The second harmonic alterations we found here and in our previous study (Vilidaite et al., 2018) that compared human ASD responses with a genetic ASD model provide evidence for two of four previously proposed core phenotypes for ASD—these are alterations that occur in sensory-dedicated regions of cortex and are evident in genetic animal models of the condition (Robertson & Baron-Cohen, 2017).

### Cross-modal sensory alterations

ASD and ADHD as clinical diagnoses were considered to be mutually exclusive diagnoses in the *Diagnostic and Statistical Manual of Mental Disorders, Fourth Edition* (DSM-IV). However, comorbidity is common between ASD and ADHD on a range of cognitive, social, and motor functions (Rommelse, Franke, Geurts, Hartman, & Buitelaar, 2010; van Steijn et al., 2012), and this restriction was lifted in DSM-V. In this present investigation, we composed our ASD and ADHD groups on the basis of a comprehensive clinical characterization that included the administration of the ADOS-2, ADI-R, and K-SADS-PL instruments. Having made diagnostic distinctions on this basis, we found both distinct (as just described) and shared alterations of sensory evoked responses in the two disorders.

### Shared alterations in cross-modal interaction

A previous fMRI study also found that downregulation of responses in early visual cortex by auditory stimuli is altered in ASD (Keehn et al., 2017). Here, we identified a similar pattern, such that an increasing sound-level auditory stimulus causes a reduction in the visual 1F1 response. This response reduction, however, was absent in both ASD and ADHD groups and is thus a shared neural phenotype in our paradigm. The cross-modal interaction we observe in TD children could be mediated by direct projections from auditory to visual cortex that are known to exist in primates (Falchier, Clavagnier, Barone, & Kennedy, 2002; Majka et al., 2019; Rockland & Ojima, 2003). These connections could mediate direct suppression via inhibition or they could mediate attentional diversion from the visual to auditory domains in TD children (Murray, Thelen, Thut, Romei, Martuzzi, & Matusz, 2016; Petro, Paton, & Muckli, 2017). The alterations seen in the ASD and ADHD groups could be either anatomical (weakening of the projection) or functional. An influence of visual stimulation on the auditory response was measurable in all groups when the auditory stimulus was of high intensity. This suggests that alterations in A/V interaction are not symmetric, as the influence of auditory stimulation on visual responses was altered in both ASD and ADHD, but the influence of visual stimulation on auditory responses was not, at least within the limits of our measurements.

### Mechanisms of ASD and ADHD sensory alterations

As noted in the Introduction, ASD sensory processing alterations have been suggested to be the result of alterations in the balance of excitation and inhibition (Rosenberg et al., 2015; Rubenstein & Merzenich, 2003; Yizhar et al., 2011). Beyond simply measuring explicit hyperresponsivity, the shape of the contrast response function provides additional information about possible modifications in the underlying sensory mechanisms. The relative lack of saturation of the 1F1 contrast response provides at least partial support for the E/I imbalance theory in that the typically observed saturation of the visual response at high contrasts was less prominent in both the ASD and ADHD groups. Whether this alteration has behavioral consequences remains to be determined.

A general E/I imbalance would, however, predict that the alteration should also be present at 2F1, which was not the case in our measurements. The differential effects of ASD and ADHD on the shape of the 1F1 and 2F1 contrast response functions and the selective loss of second-harmonic amplitude in ASD more generally indicate that these two response components arise at least partially from separate neural substrates. The pattern of alteration of first versus second harmonics suggests a pathway-based model in which the transient visual pathway is selectively altered in ASD in early visual cortex (Vilidaite et al., 2018). The observation that blood oxygenation level-dependent (BOLD) responses in V1 to a coherent motion stimulus are altered in ASD (Robertson, Thomas, Kravitz, Wallace, Baron-Cohen, Martin, & Baker, 2014) is consistent with this view, given that such responses are thought to rely on magnocellular inputs. These alterations occur at least by the level of early visual cortex but could be present in the subcortical afferents arising from the retina. There is mounting evidence for precortical alterations in the sensory pathways of persons with ASD (Dadalko & Travers, 2018), with the best evidence for thalamocortical alterations in ASD coming from studies of auditory brainstem responses (Miron, Beam, & Kohane, 2018). Evidence for a similar modification in visual thalamic responses is currently lacking, but the earliest latency transient VEP responses have been reported to be reduced in amplitude in ASD (Siper et al., 2016). Interestingly, another potential pathway-related mechanism may exist in ADHD, given that VEPs to chromatic stimuli modulated along the blue/yellow color axis have been found to be altered (Kim et al., 2015). These stimulation conditions favor activation of the koniocellular pathway through the geniculate. The koniocellular, magnocellular, and parvocellular pathways form the main inputs from the retina to visual cortex (Hendry & Reid, 2000). Further studies using pathway-isolating stimuli and analysis procedures, as well as direct neural measurements, will be useful in fully understanding the initial locus of sensory processing alterations in developmental disorders such as ASD and ADHD.

### Limitations

The primary goal of the present study was to compare sensory responses in ASD and TD groups. We included an ADHD group in order to assess the specificity of the effects observed to ASD, as opposed to effects that are shared with other developmental disorders. A limitation of our study is the likelihood of comorbidity of ASD and ADHD, which could have minimized differences between the groups. We used clinical diagnostic criteria to make the classification, but more sophisticated phenotypic assessments may have provided better separation of the two groups. Nonetheless, in making this comparison, we found qualitative differences between the ASD and ADHD groups that manifested primarily as selective loss of transient responses in ASD and a common loss of A/V interaction.

The sample size for the ADHD group was small relative to the ASD and TD groups, potentially limiting the generalizability of these conclusions if the ADHD group we sampled was not representative. The design of our study allowed for multiple internal consistency checks on the effects based on independent recordings. The main effects were repeatable, and this suggests that sample size was not a limiting factor in detecting the effects we report here. Moreover, the pattern of loss seen in the present ASD sample replicates the pattern we observed previously in a large sample of adults with elevated AQ and smaller samples of adult and child ASD participants (Vilidaite et al., 2018).

Visual acuity for contrast-modulated gratings is adult-like by 6 years (Skoczenski & Norcia, 1999), the age of the youngest group we have tested, but the specific nature of alterations in responsiveness may nonetheless depend on the age of the participants at the time of testing. Alterations of VEPs to simple patterns do persist into adulthood (see Table 1). Our previous work with a fly model of ASD found that the alterations of response dynamics differed between adult and juvenile flies tested on the same protocol, with adult flies showing second-harmonic response reductions and immature flies showing first-harmonic reductions. Previous research has additionally suggested amelioration of A/V integration differences in ASD by adolescence (Beker et al., 2018). There have been no studies of sensory evoked responses in human ASD that have spanned both children and adults using the same response measures. Such a study would be necessary to address specifically developmental versus generic alterations of sensory processing that are stable across age.

**Figure 6.**
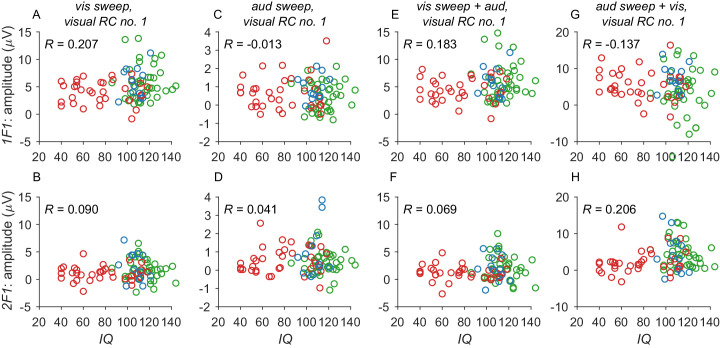
Visual response projected amplitudes versus SB-5 IQ score for stimulus-related frequencies 1F1 (top row) and 2F1 (bottom row) derived from visual RC1. Data from TD children are shown in green, ASD children in red, and ADHD in green. Plotting is as in Figure 2. Unimodal visual response correlations are shown in (A) for 1F1 and (B) for 2F1. Background EEG correlations during unimodal auditory stimulation are shown in (C) and (D) for 1F1 and 2F1, respectively. Visual response correlations measured in the presence of a highly supra-threshold auditory input are shown in (E) and (F) for 1F1 and 2F1, respectively. Visual response correlations for a 40% contrast grating measured in the presence of a variable sound-level auditory input are shown in (G) and (H) for 1F1 and 2F1, respectively. Correlation values are shown as insets.

The SB-5 measure of IQ was not matched across groups, and this could have contributed to group-level differences in the EEG measures. A control analysis was therefore performed to test for the possibility that our results were driven by differences in IQ among our participant groups. For each of the eight conditions shown in Figure 2, we created a single summary score for each child's response function. This summary score was the average amplitude over the 10 response bins we measured for the 1F1 or 2F1 responses. We then correlated these eight summary scores with IQ. As can be seen in Figure 6, the maximal correlations were around 0.2 (e.g., they would account for ∼4% of the variance; range, –0.013 to 0.207). IQ does not measurably moderate our evoked potential responses on this analysis. This is not to say that effects might be seen in a larger sample or with more a more complex analysis or different measurement modality. For example, measurements of GABA concentrations in visual cortex have been found to correlate with IQ (Cook, Hammett, & Larsson, 2016), with persons with higher IQ having higher gamma aminobutyric acid concentrations. Tonic levels of GABA could alter the patterns of cross-modal suppression we observed in ASD.

Another limitation of our study is that we have assessed auditory, visual, and auditory–visual responsiveness over a limited range of temporal and spatial parameter values. The use of single-frequency stimulation within a sensory modality, although necessary for implementation of the frequency-tagging approach, yields stimuli that are not as complex as natural stimulation, and they probe the system over only a limited range of inputs. Moreover, the fact that both auditory and visual systems are nonlinear limits generalizing our results to more complex stimuli. Additional recordings over a wider range of stimulus conditions are necessary in order to assess the generalizability of our findings to other stimulus conditions.

## Conclusions

Children with ASD and ADHD have alterations in visual, auditory, and A/V responses suggestive, on one hand, of common mechanistic alterations in A/V interaction and, on the other, disjoint effects on transient versus sustained visual processes. The altered transient responses in ASD are likely to arise very early in the visual pathway and could thus have downstream consequences for many other visual mechanisms and processes. The shared alteration in A/V interaction could be a signature of a comorbid phenotype shared by ASD and ADHD, possibly due to alterations in attentional selection systems.

## Supplementary Material

Supplement 1

Supplement 2
