# Supplementary material for: A case–control study of visual, auditory and audio–visual sensory interactions in children with autism spectrum disorder
Source: J Vis. 2021 Apr 8;21(4):5. doi: 10.1167/jov.21.4.5 (PMC8039569; doi:10.1167/jov.21.4.5)
Supplement: Supplement 1 [file jovi-21-4-5_s001.pdf]

Supplementary materials 1

Full Clinical Diagnostic Criteria Battery

In addition to the ADOS-2, ADI-R, and K-SADS administered for participant group inclusion or exclusion as described in the Methods section, each participant also completed the Stanford Binet Intelligence Scales, 5th Edition (SB-5; Roid, 2003), and parents/legal guardian filled out the Sensory Profile Caregiver Questionnaire (SPQ; Dunn, 1999), the Child Behavior Checklist (CBCL; Achenbach, 2001), the Social Responsiveness Scale, 2nd Edition (SRS-2); the Repetitive Behavior Scales, Revised (RBS-R; Lam & Aman, 2007), and the Vineland Adaptive Behavior Scales, 2nd Edition (VABS-2; Sparrow et al, 2005). The SB-5 is a standardized assessment measuring intellectual quotient (IQ; full scale, verbal, and nonverbal) in individuals aged 2 years or older. The SPQ is a 125-item caregiver questionnaire that measures behaviors in response to events in multiple domains of sensory processing, including visual, auditory, taste, smell, touch, body position, movement, and socio-emotional processing. The RBS-R is a 43-item caregiver questionnaire that rates repetitive behaviors. The VABS-2 is a standardized rating scale that measures development and adaptive behavioral functioning, such as communication, socialization, daily living skills, and motor skills.

Inclusion/Exclusion Criteria

ASD group. Participants who met DSM-5 criteria, determined by a clinical evaluation and confirmed by the ADI-R and ADOS2 were initially deemed members of the ASD group. Participants additionally needed to be capable of participating in all testing procedures, including interviews and EEG recordings, insofar as valid, standard scores could be obtained. If participants were on medications (N = 7), they had to be stable for at least one week prior to EEG recording. Participants were excluded if there were evidence of genetic, metabolic, or infectious etiologies for their ASD on the basis of medical history, neurological history, as well as laboratory testing for inborn errors of metabolism and chromosomal analysis when indicated. Any child with a severe mental disorder, such as schizophrenia or bipolar disorder, or an unstable medical condition, like seizures or heart disease, were also excluded.

ADHD group. Participants determined to meet DSM-5 criteria for ADHD during a clinical interview and confirmed with the K-SADS were accepted for inclusion to the ADHD group. Children additionally needed to demonstrate clinical hyperactivity and/or inattention measures on subscales of the Child Behavior Checklist (CBCL). Participants needed to be medically healthy, free of neurological disorders and have no significant hearing or vision impairments. If participants were on

medications, they needed to be stable for at least one week prior to EEG recording. Children were excluded if they had a first-degree relative with ASD or if they personally had a known medical condition, like seizures or heart disease. Children scoring over 60 on the SRS-2 were also excluded in order to minimize co-morbidity with ASD

TD group. Participants in the NT needed to be medically healthy, without any significant hearing or vision impairments. Children were included in the NT group as long as valid results from all standard procedures could be collected. Children who demonstrated past or present neurological by history or psychiatric disorders on the CBCL or K-SADS were excluded. If parents experienced significant difficulty during pregnancy, labor, delivery, or the immediate neonatal period, or if the participant experienced abnormal milestones, the child was excluded.
