# Supplementary material for: A case–control study of visual, auditory and audio–visual sensory interactions in children with autism spectrum disorder
Source: J Vis. 2021 Apr 8;21(4):5. doi: 10.1167/jov.21.4.5 (PMC8039569; doi:10.1167/jov.21.4.5)
Supplement: Supplement 2 [file jovi-21-4-5_s002.pdf]

Supplementary Data: Demographics and clinical scores

ASD	*Full Scale IQ*	*Age (months)*	*Age (years)*	*Gender*	*ADOS Score*
1	54	79.1	6.6	Male	10
2	40	81.6	6.8	Male	NA
3	80	59.2	4.9	Male	8
4	112	65.9	5.5	Male	8
5	40	71.4	6.0	Female	10
6	42	65.9	5.5	Male	9
7	104	88.6	7.4	Male	6
8	75	51.2	4.3	Male	7
9	76	102.8	8.6	Male	9
10	118	83.8	7.0	Male	7
11	98	77.3	6.4	Male	9
12	50	87.4	7.3	Female	6
13	104	62	5.2	Female	8
14	54	95.3	7.9	Male	6
15	60	74.1	6.2	Female	8
16	112	111.7	9.3	Female	5
17	60	49.5	4.1	Male	7
18	78	56.4	4.7	Male	8
19	52	112.3	9.4	Male	9
20	104	114.1	9.5	Male	7
21	54	98.8	8.2	Male	6
22	75	119.8	10.0	Female	4
23	58	71.5	6.0	Male	8
24	86	65.2	5.4	Male	8
25	40	97.5	8.1	Male	6
26	113	110.9	9.2	Male	NA
27	113	117.9	9.8	Male	9
28	108	74.2	6.2	Male	7
29	66	69.9	5.8	Male	6
30	87	66.5	5.5	Male	NA
31	68	112.7	9.4	Male	10
32	78	95.5	8.0	Male	7
33	51	107.5	9.0	Male	7
34	102	50.2	4.2	Male	NA
**ASD Mean**	76.8	83.8	7.0		
**Std. Deviation**	25.4	21.6	1.8		

**TD**	Full Scale IQ	Age (months)	Age (years)	Gender
1	105	83.5	7.0	Female
2	106	62.8	5.2	Female
3	107	51.8	4.3	Male
4	135	62.6	5.2	Male
5	144	102.5	8.5	Male
6	110	53.3	4.4	Female
7	131	84.7	7.1	Male
8	119	54.5	4.5	Male
9	112	89.5	7.5	Male
10	103	92.1	7.7	Male
11	110	51.4	4.3	Male
12	111	80.3	6.7	Female
13	121	110	9.2	Female
14	127	85.4	7.1	Male
15	116	63.1	5.3	Female
16	118	79.5	6.6	Male
17	130	80.2	6.7	Male
18	127	81.1	6.8	Male
19	98	78.4	6.5	Male
20	120	104	8.7	Male
21	86	77.7	6.5	Male
22	124	75.1	6.3	Male
23	141	68.1	5.7	Male
24	98	109.2	9.1	Female
25	102	66.4	5.5	Male
26	112	94	7.8	Male
27	120	67.9	5.7	Male
28	108	69.9	5.8	Male
29	114	64.2	5.4	Male
30	104	65.9	5.5	Male
31	111	65.9	5.5	Male
32	125	67.7	5.6	Male
33	122	69.8	5.8	Male
34	101	70.5	5.9	Male
35	118	63.9	5.3	Male
36	121	64	5.3	Male
37	111	65.7	5.5	Male
38	110	62.9	5.2	Male

39	124	93.7	7.8	Female
40	110	113.9	9.5	Male
**TD Mean**	115.3	76.2	6.3	
**Std. Deviation**	12.0	16.5	1.4	

**ADHD**	Full Scale IQ	Age (months)	Age (years)	Gender
1	114	100.9	8.4	Male
2	112	117.6	9.8	Female
3	111	90.1	7.5	Male
4	121	101.4	8.5	Male
5	114	100.4	8.4	Male
6	106	104.3	8.7	Female
7	97	116.5	9.7	Male
8	109	89.3	7.4	Male
9	99	107.6	9.0	Male
10	92	89.3	7.4	Male
11	105	80.2	6.7	Male
12	104	67	5.6	Male
13		117	9.8	Male
**ADHD Mean**	107.0	98..6	8.2	
**Std. Deviation**	8.2	14.7	1.3	
